# Grazing Affects Exosomal Circulating MicroRNAs in Cattle

**DOI:** 10.1371/journal.pone.0136475

**Published:** 2015-08-26

**Authors:** Susumu Muroya, Hideki Ogasawara, Masayuki Hojito

**Affiliations:** 1 Animal Products Research Division, National Institute of Livestock and Grassland Science, Tsukuba, Ibaraki, Japan; 2 Field Science Center, School of Veterinary Medicine, Kitasato University, Yakumo, Hokkaido, Japan; University of Massachusetts Medical, UNITED STATES

## Abstract

Circulating microRNAs (c-miRNAs) are associated with physiological adaptation to acute and chronic aerobic exercise in humans. To investigate the potential effect of grazing movement on miRNA circulation in cattle, here we profiled miRNA expression in centrifugally prepared exosomes from the plasma of both grazing and housed Japanese Shorthorn cattle. Microarray analysis of the c-miRNAs resulted in detection of a total of 231 bovine exosomal miRNAs in the plasma, with a constant expression level of let-7g across the duration and cattle groups. Expression of muscle-specific miRNAs such as miR-1, miR-133a, miR-206, miR-208a/b, and miR-499 were undetectable, suggesting the mildness of grazing movement as exercise. According to validation by quantitative RT-PCR, the circulating miR-150 level in the grazing cattle normalized by the endogenous let-7g level was down-regulated after 2 and 4 months of grazing (*P* < 0.05), and then its levels in housed and grazing cattle equalized when the grazing cattle were returned to a housed situation. Likewise, the levels of miR-19b, miR-148a, miR-221, miR-223, miR-320a, miR-361, and miR-486 were temporarily lowered in the cattle at 1 and/or 2 month of grazing compared to those of the housed cattle (*P* < 0.05). In contrast, the miR-451 level was up-regulated in the grazing cattle at 2 months of grazing (*P* = 0.044). The elevation of miR-451 level in the plasma was coincident with that in the *biceps femoris* muscle of the grazing cattle (*P* = 0.008), which suggests the secretion or intake of miR-451 between skeletal muscle cells and circulation during grazing. These results revealed that exosomal c-miRNAs in cattle were affected by grazing, suggesting their usefulness as molecular grazing markers and functions in physiological adaptation of grazing cattle associated with endocytosis, focal adhesion, axon guidance, and a variety of intracellular signaling, as predicted by bioinformatic analysis.

## Introduction

Grazing is being promoted over pen confinement to improve animal welfare, and potentially has an influence on the physiology of cattle and hence beef quality by the intermittent movements involved. In Holstein and Japanese black cattle, grazing was found to affect metabolic properties and the fiber-type composition of their skeletal muscles [1, 2]. The oxidative properties of muscle in grazing Charolais steers was shown to be altered as a result of increased mobility and a grass (vs. maize silage) diet on pasture [3]. Activities of oxidative enzymes, isocitrate dehydrogenase and citrate synthase, were found to be higher in muscles from grazing animals, demonstrating a plasticity of muscle metabolism according to the production and feeding system [4]. To improve cattle production system for the better animal welfare and beef quality, it is essential to explore biomarkers to distinguish extensive beef production.

In grazing cattle, microRNAs (miRNAs) are expected to modulate gene expression in muscles, as grazing affects muscle properties. MiRNAs are highly conserved, noncoding small RNAs that regulate the expression of target genes in various biological processes, including myogenesis. Transcribed pri-miRNAs are processed into pre-miRNAs and finally into mature miRNAs that recognize target genes as components of the RNA-induced silencing complex (RISC), resulting in mRNA degradation or destabilization. Recent studies of muscle-specific miRNAs such as miR-1, miR-133a/b, miR-206, and miR-208b have indicated their roles in the development or specification of skeletal muscle [5–7]. Some miRNAs are suggested to mediate muscle adaptations in response to exercise [8–10], immobilization [11], and amino acid intake as diet [12], as well as muscle disorders [13–15]. We recently reported that miR-206 and miR-208b expressions in the *biceps femoris* (BP) muscle of grazing Japanese Shorthorn cattle were elevated after 4 months of grazing; these miRNAs are associated with temporary down-regulation of MyoD and fast-type myosin heavy chain isoform [16], which could be associated with conversion of the bovine skeletal muscle type with miRNA profile [17].

MiRNAs could be released from a variety of tissues in response to the phenotypic changes for physiological adaptation [15]. It has been well studied, especially regarding disease, that some miRNAs are secreted from cells into circulation or taken from circulation into cells. Serum levels of miR-1, miR-133a/b, and miR-206 are increased in patients of human Duchenne muscular dystrophy [18, 19] and of rhabdomyosarcoma tumor [20], and that the miR-21 level is affected in various types of cancers [21–24]. Furthermore, the circulating level of miR-144 is increased in type 2 diabetic humans as well as rat model, and this elevation is negatively correlated with insulin receptor substrate 1 in insulin-responding tissues including skeletal muscles [25]. Besides serum and plasma, growing evidence has shown that miRNAs are present in various human body fluids including breast milk, urine, and saliva. A large part of those circulating miRNAs (c-miRNAs) are packed in extracellular microvesicles with diameters ranging from 30 to 100 nm, namely exosomes [26], which thereby make them resistant to low pH and RNase degradation [27] and available as potential diagnostic biomarkers.

C-miRNAs are also associated with physiological conditions in exercise as well as pathology [28]. A single bout of exhaustive cycling or rowing training for 90 days elevated plasma miR-20a, miR-21, miR-146a, miR-221, and miR-222 levels [29], whereas a single bout of cycling exercise at 70% VO_2 max_ for 60 min decreased the circulating level of miR-486 immediately after the exercise [30]. Moreover, circulation levels of miR-1, miR-133a/b, and/or miR-208b significantly increased during early recovery of muscle-damaging downhill walking in healthy subjects [31] and in the 3-h period following a non-muscle-damaging single bout of cycling [32], suggesting that the profile of c-miRNAs are affected by the mode and intensity of the exercise.

Diet and environmental stress also potentially cause changes in physiological conditions of cattle during grazing. C-miRNAs could be useful biomarkers to characterize grazing cattle with higher welfare and beef quality. In the present study, we hypothesized that grazing, accompanied by alteration of physiological conditions, would affect the c-miRNA profile in cattle and thereby could be characterized by specific c-miRNAs as biomarkers. The objective of this study was to identify exosomal c-miRNAs that are changed in cattle by grazing on pasture, to elucidate the roles of c-miRNAs in the physiological adaptation and muscle formation of grazing cattle.

## Results

### RNA composition in bovine plasma exosomes

**Fig 1:**
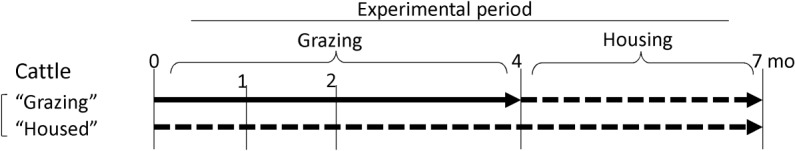
Scheme of the experimental period for investigation of gene expression in grazing JSH cattle. Solid and broken lines indicate grazing and housing of cattle, respectively.

**Fig 2:**
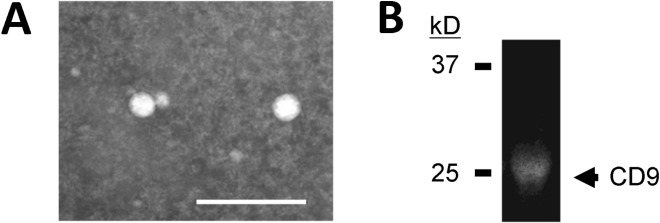
Properties of bovine plasma exosomes isolated by ultracentrifugation. (A) Representative transmission electron microscopic images of bovine plasma exosomes. The bar indicates 200 nm. (B) Detection of CD9 protein in bovine plasma exosomes by immunoblotting.

**Fig 3:**
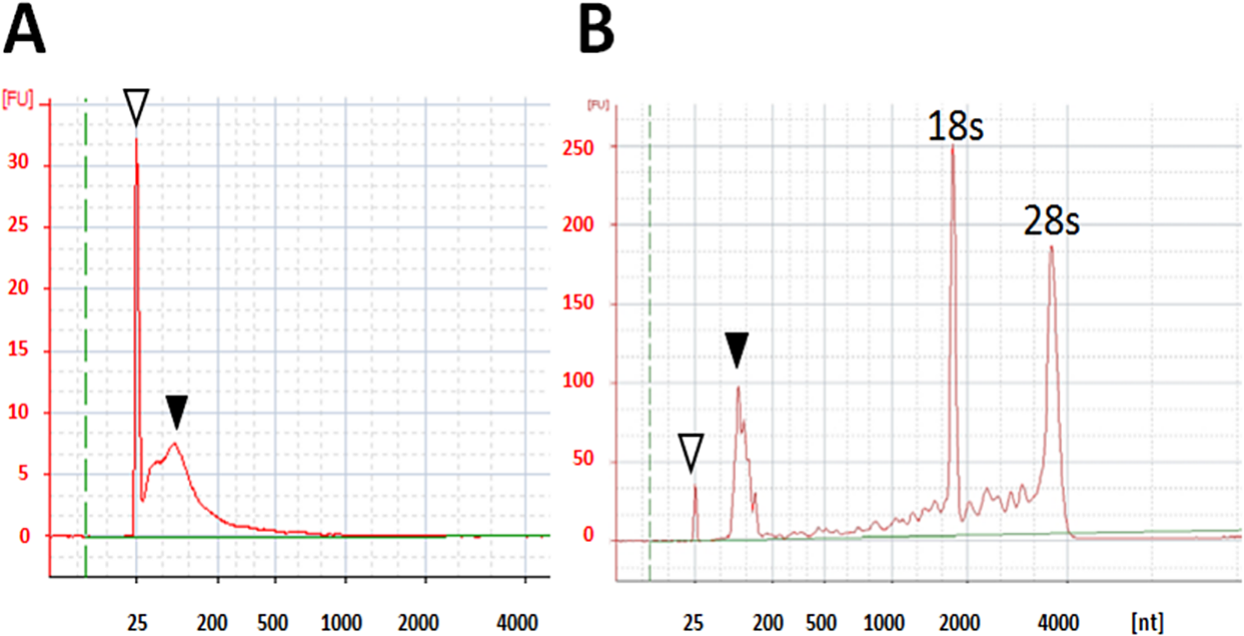
Representative electropherogram of RNA samples prepared from bovine plasma exosome (A) and skeletal muscle (B) Open and closed triangles indicate peak front and small RNA, respectively.

Animal management and sample collection were conducted as illustrated in Fig 1. In the present study, plasma exosomes of grazing cattle were prepared for analysis of c-miRNAs in exosomes without cellular RNAs by an ultracentrifugal method based on a method of Rani *et al* [33], and the c-miRNAs in the plasma samples were successfully profiled. Representative plasma exosomes were globular and approximately 60 nm in diameter as shown in Fig 2A, and contained a CD9 protein known as an exosome marker (Fig 2B). The total RNA samples prepared from the plasma exosomes exclusively consisted of small RNAs, but did not include ribosomal 18s and 28s or other long RNAs such as mRNAs, whereas total muscle RNAs contained ribosomal 18s and 28s RNAs and mRNAs as major components (Fig 3).

### Plasma exosomal miRNA profiles of grazing cattle

**Fig 4:**
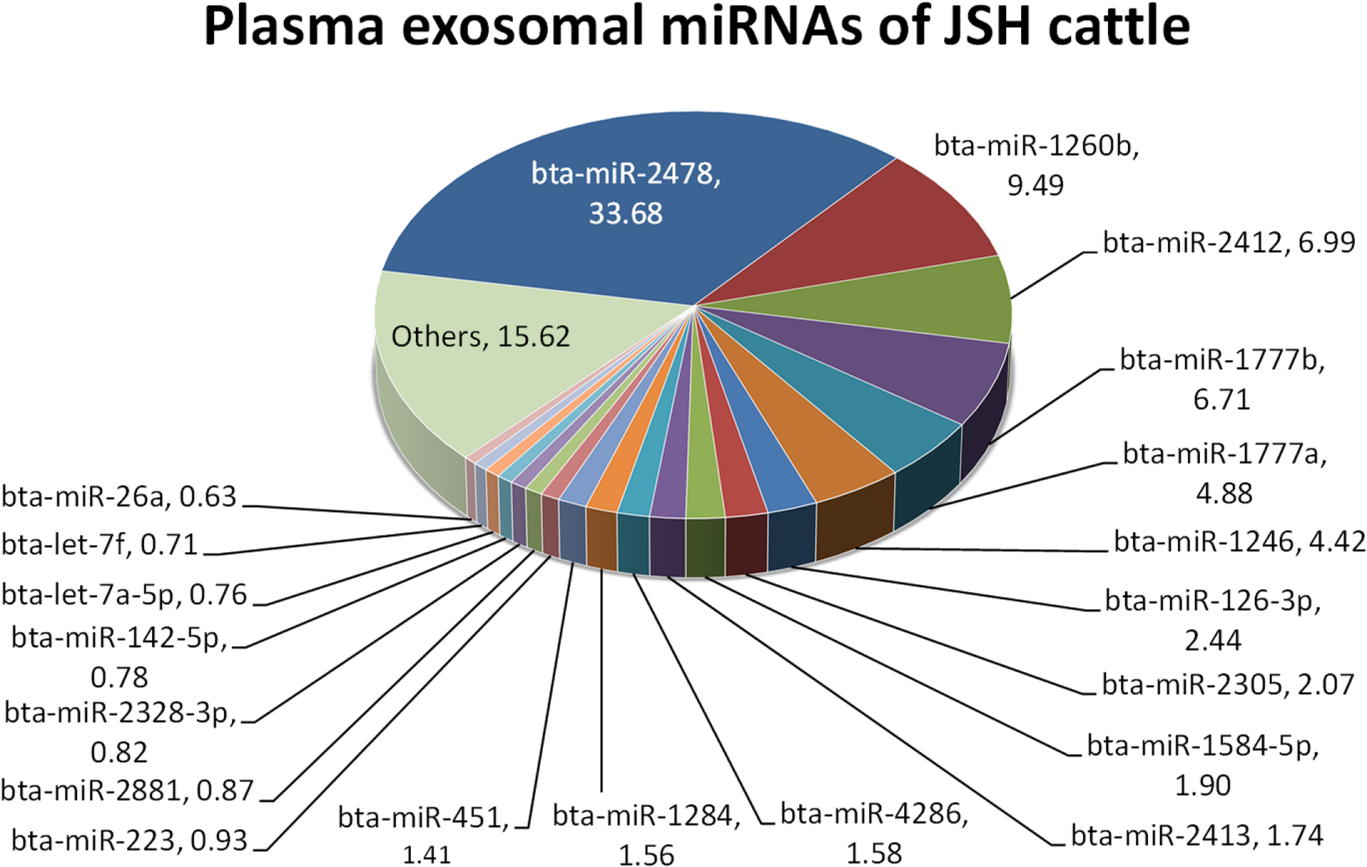
MicroRNA composition of bovine plasma exosome. MicroRNA (miRNA) names and percentages of total miRNA contents are indicated.

Using the plasma exosomal RNA mixture of 3 steers for each time point in the grazing or housed group, we conducted microarray analysis of the plasma exosomal c-miRNAs in cattle, to overview grazing-induced changes in the c-miRNA profile (S1 Table). Totally 231 miRBase-registered bovine miRNAs were detected in the exosome samples. The c-miRNA profile of the housed cattle at time 0 showed that the top 20 miRNAs comprised 84.38% of the total miRNAs, of which the most and second-most abundant were miR-2478 (33.68%) and miR-1260b (9.49%), respectively (Fig 4). Also present were miR-1777b (6.71%), miR-1777a (4.88%), miR-1246 (4.42%), miR-126-3p (2.44%), miR-2305 (2.07%), miR-1584-5p (1.90%), miR-2413 (1.74%), miR-4286 (1.58%), miR-1224 (1.56%), and miR-451 (1.41%). Muscle-specific miRNAs such as miR-1, miR-133a, miR-206, miR-208b, and miR-499 were not significantly detected in the plasma exosomes across all samples (i.e., grazing and housed during experiment) except for miR-486 (0.18%) and a trace of miR-133b (< 0.001%).

Of the 231 c-miRNAs, 131 miRNAs with a high level of raw signal intensity (> 30.0) and insignificant difference between cattle groups at time 0 were applied to hierarchical clustering. Among those miRNAs, the normalized expression level of let-7g, which employed a mixture of samples from 3 grazing or housed cattle for each time point, was stable in each cattle group during the experimental period without apparent difference between the cattle groups (S1 Fig). Therefore, we employed the let-7g level as the normalizing factor for further qRT-PCR analysis in this study.

**Fig 5:**
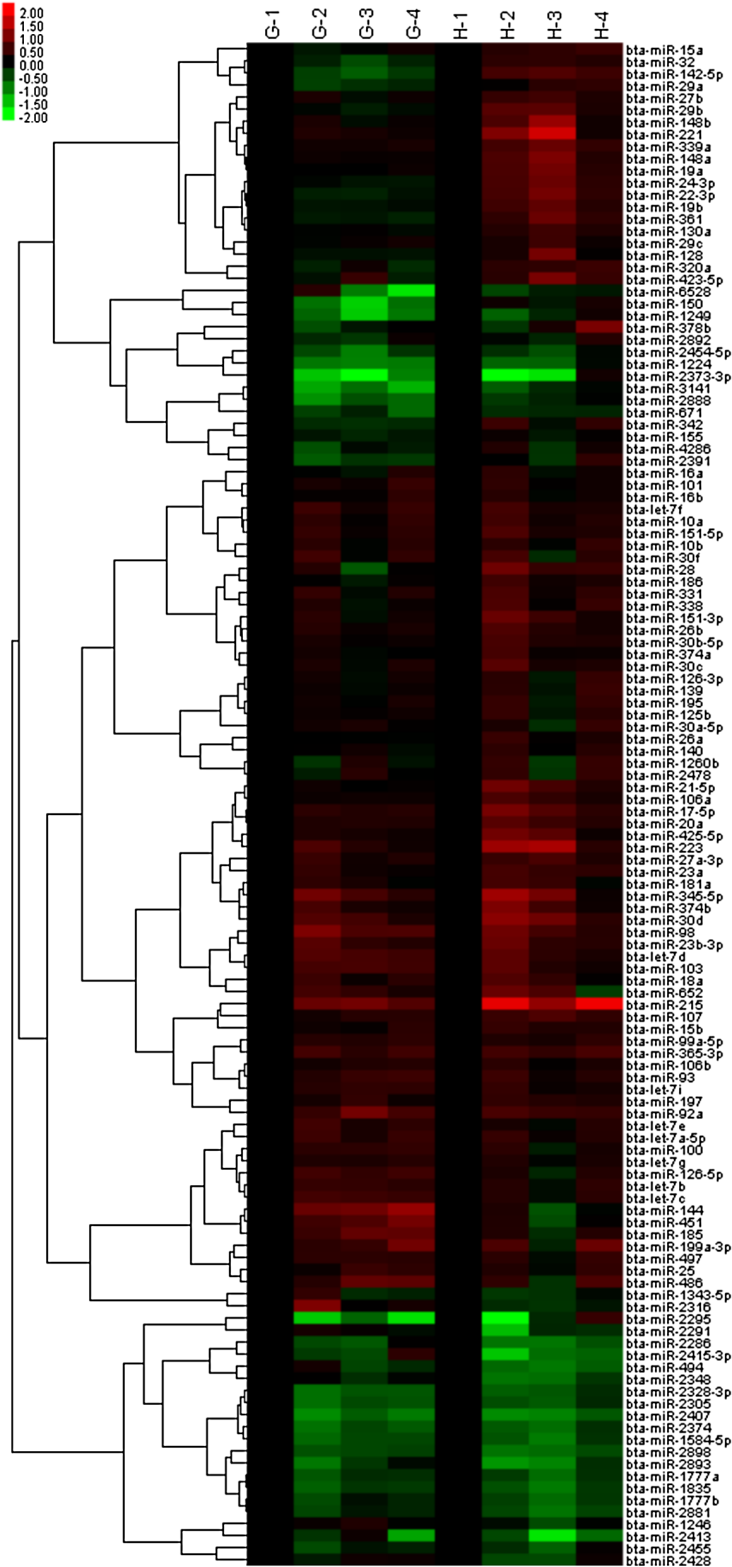
Hierarchical clustering result of microarray analysis for 131 exosomal miRNAs in plasma of grazing and housed cattle. The hierarchical clustering was performed using log-transformed values of miRNA expression levels at 0, 1, 2, and 4 mo in plasma exosome samples of grazing cattle (G-1, G-2, G-3, G-4) and (H-1, H-2, H-3, H-4). The values of G-2, G-3, and G-4 samples were normalized by that of G-1, while values of H-2, H-3, and H-4 samples were by that of H-1.

A heatmap of the 131 c-miRNAs showed that these miRNAs can be clustered into primarily 4 clusters according to the changes in their levels in grazing and housed cattle during the first 4 months (duration of grazing): (1) increase in both cattle groups, (2) decrease in both cattle groups, (3) increase in the grazing and decrease in the housed, (4) decrease in the grazing and increase in the housed (Fig 5). In this overview, it was noted that the only miRNAs that were observed to increase in the grazing but not in the housed cattle were miR-144 and miR-451, whereas there were many more miRNAs with an increasing tendency in the housed but not in the grazing, such as miR-150 and miR-223.

### Quantitative changes in circulating levels of exosomal miRNAs

Of the 131 miRNAs, we further selected several 20 miRNAs that appeared to differ significantly between cattle groups, and verified the changes in their circulation levels quantitatively. In the qRT-PCR analysis, equal amounts of plasma samples among all animals and time points were used for preparation of exosomal RNA. No difference in the level of circulating let-7g was observed between cattle groups or among time points (*P* > 0.10).

**Fig 6:**
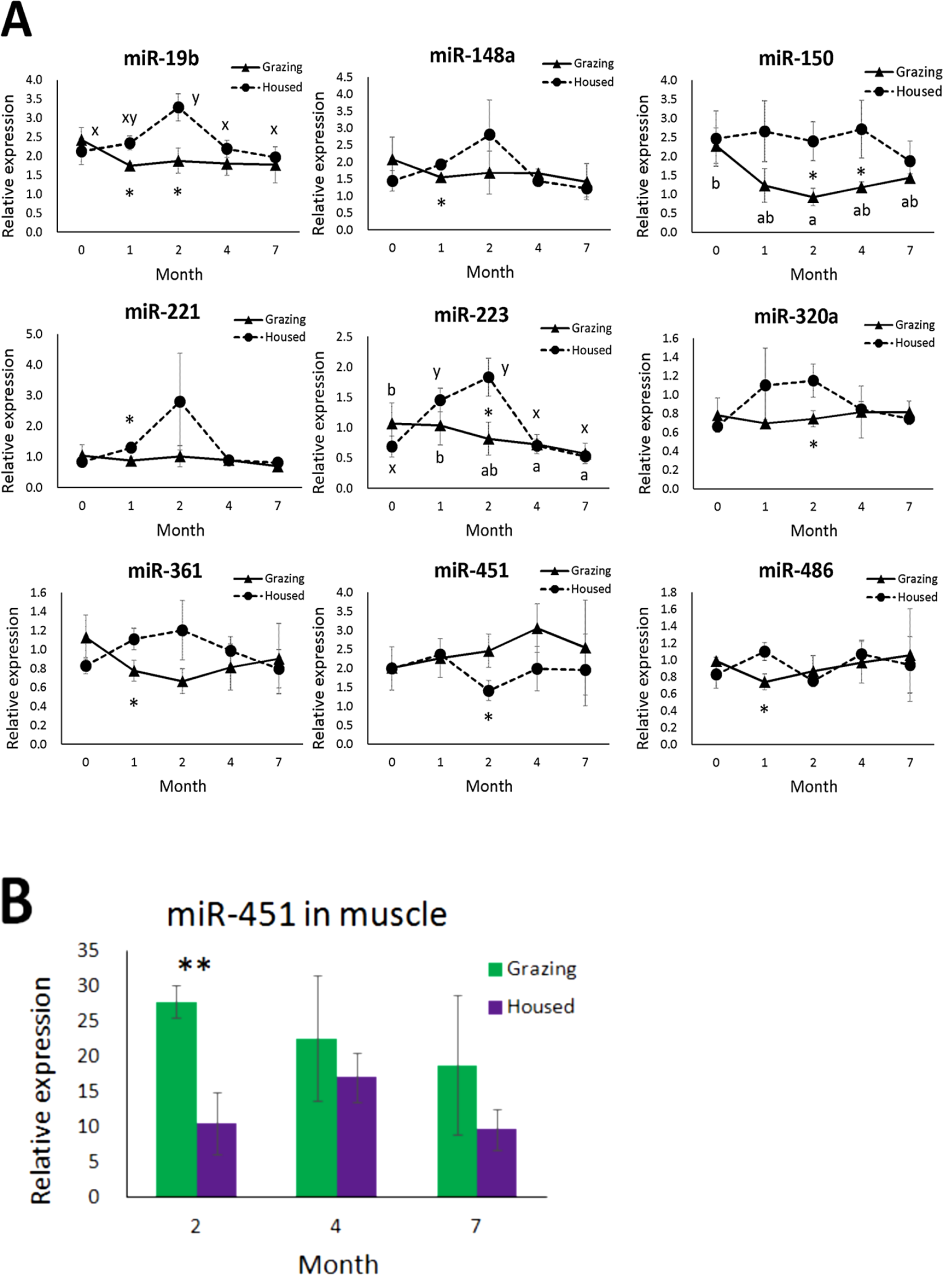
Changes in levels of 9 plasma exosomal miRNAs (A) and muscular miR-451 (B) of grazing and housed cattle analyzed by qRT-PCR. Data of grazing and housed cattle are indicated as solid and broken lines in panel A, and green and purple columns in panel B, respectively. Different letters among the time points indicate differences at *P* < 0.05 in the grazing (x, y) and housed cattle (a, b). An asterisk and double asterisks indicate differences between the cattle groups at *P* < 0.05 and *P* < 0.01, respectively.

The results of qRT-PCR normalized with the let-7g level showed that the levels of miR-19b, miR-148a, miR-150, miR-221, and miR-361, and miR-486 in the grazing cattle were lower than those in the housed cattle at 1 mo of grazing (*P* = 0.013, 0.014, 0.093, 0.011, 0.041, and 0.023, respectively) (Fig 6A). At 2 mo, the levels of miR-19b, miR-150, miR-223, miR-320a, and miR-361 in the grazing cattle were lower than in the housed cattle (*P* = 0.015, 0.020, 0.026, 0.023, and 0.089, respectively). The higher miR-150 level in circulation in the housed than in the grazing cattle persisted at 4 mo (*P* = 0.049). The miR-150 level in the housed cattle did not change during the experimental period, whereas that in the grazing cattle decreased from time 0 to 2 mo (*P* = 0.005). In contrast, the miR-19b level in the housed but not in the grazing cattle increased from time 0 to 2 mo (*P* = 0.006). The miR-223 level in the housed cattle increased from time 0 to 1 or 2 mo (*P* < 0.008), while in the grazing cattle, it decreased from 0 to 4 and 7 mo (*P* < 0.006).

Among the miRNAs tested, miR-451 was unique in that the circulation level in the grazing cattle was higher than in the housed cattle at 2 mo (*P* = 0.044). Since the expression level of miR-451 in skeletal muscle cells is associated with myogenesis [34], ageing [35], and exercise [36], the miR-451 expression in muscle during grazing was analyzed. Intriguingly, the miR-451 level in the BP muscle of the grazing cattle at 2 mo was also higher than that in the housed (*P* = 0.008; Fig 6B).

At 7 mo, no difference in circulation level of miRNAs was observed between the two cattle groups. Although circulation levels of miR-29b, miR-30a, miR-30d, miR-103, miR-126-5p, miR-144, miR-155, miR-425-5p, miR-489, miR-1249, and miR-2888 were also examined, no significant differences in these miRNAs were observed between the cattle groups at any time points or between the time points in either of the cattle groups.

### Prediction of biological events associated with the relevant circulating microRNAs

We further analyzed molecular biological events of the predicted target genes of the c-miRNAs with significant differences between grazing and housed cattle by functional annotation with the Database for Annotation, Visualization, and Integrated Discovery (DAVID), to characterize grazing by molecular biological phenomena in the cattle. According to the results of TargetScan analysis, totally 2743 bovine genes were predicted as the targets of c-miRNAs significantly down-regulated by grazing (miR-19b, miR-148a, miR-150, miR-221, miR-223 miR-320a, miR-361, and miR-486). Of those target genes, 2318 genes valid in the DAVID system were further applied to the functional annotation analysis, which resulted in extraction of the over-represented pathways in the Kyoto Encyclopedia of Genes and Genomes (KEGG) database such as focal adhesion, axon guidance, endocytosis, and signaling pathways related to Wnt, MAPK, neurotrophin, ErbB, insulin, TGFβ, and mTOR (Bonferroni-adjusted *P* < 0.05, Table 1). With the KEGG description, the results indicate that the predicted target genes are associated with intake of essential substances to the cell interior, signaling of extracellular growth factor ligands to intracellular signaling pathways via adhesive proteins coupled with protein kinase, intracellular signaling pathways related to mTOR, ErbB, and MAPK, possibly for Ca^2+^-sensitive muscle cell hypertrophy and force generation/transmission. TargetScan predicted 22 genes including titin as the targets of the only up-regulated c-miRNA, miR-451; however, no significant gene ontology (GO) or KEGG pathway terms were obtained.

**Table 1:** KEGG pathways associated with potential target genes of down-regulated circulating microRNAs in grazing cattle.

KEGG Pathway	Number of potential target genes involved	Bonferroni *P* *
bta04510:Focal adhesion	55	7.28E-07
bta04360:Axon guidance	40	1.48E-06
bta04310:Wnt signaling pathway	46	1.62E-06
bta05200:Pathways in cancer	77	2.27E-06
bta05211:Renal cell carcinoma	27	1.33E-05
bta04010:MAPK signaling pathway	66	2.00E-05
bta04722:Neurotrophin signaling pathway	38	4.72E-05
bta05212:Pancreatic cancer	26	1.15E-04
bta04916:Melanogenesis	31	1.16E-04
bta05214:Glioma	24	1.85E-04
bta04012:ErbB signaling pathway	28	2.33E-04
bta05220:Chronic myeloid leukemia	27	2.64E-04
bta05210:Colorectal cancer	29	3.52E-04
bta04144:Endocytosis	47	9.80E-04
bta04910:Insulin signaling pathway	36	1.79E-03
bta04350:TGF-beta signaling pathway	26	3.47E-03
bta05223:Non-small cell lung cancer	19	7.46E-03
bta04720:Long-term potentiation	21	7.74E-03
bta05215:Prostate cancer	26	1.10E-02
bta05414:Dilated cardiomyopathy	24	2.00E-02
bta04150:mTOR signaling pathway	18	3.57E-02
bta04120:Ubiquitin mediated proteolysis	34	4.84E-02

* Bonferroni-adjusted *P*-value

## Discussion

### Effects of grazing on circulating miRNA

In the present study, we investigated the effects of grazing on the expression of c-miRNAs in the plasma of cattle, with the hypothesis that the c-miRNA profile reflects the physiological adaptation of various tissues such as skeletal muscles and adipose tissue. We attempted to minimize the potential effect of nutritional differences between the grazing and housed cattle on muscle gene expression, by feeding the housed cattle with grass harvested every morning. Although the effect of nutrition or movement during grazing could not be separately evaluated, the grazing of cattle in our previous study [16] resulted in significant up-regulation of miR-208b and down-regulation of miR-206, MyoD and MyHC-2x, which indicated that the skeletal muscle of the grazing cattle physiologically adapted to the increased movement.

Notably, the present study showed that effect of grazing on c-miRNAs, such as miR-19b and miR-150, was maintained for a month or more. This means that results of the present study differ from those of human acute and endurance exercise studies in which the effect of relatively intensive and strong exercise was investigated [37], as such studies have not examined the durability of the effect on c-miRNA changes.

Since no difference between the cattle groups was observed in muscle-specific miRNAs, it was suggested that the skeletal muscle and heart were not damaged and therefore the grazing could be considered a mild exercise or movement. Inflammation-related miR-155 [38] was detected, but its circulation level did not significantly differ among cattle groups or time points. In addition, miR-146a was not detected in cattle plasma in the present study, whereas it has been reported to be affected by acute exercise or endurance training [29, 39]. There is an essential role of miR-146a in inflammatory signaling in multiple cell types via its regulation of NF-κB and IL-1 receptor-associated kinase-1 as direct targets [40]. Therefore, an inflammatory response might not be elevated in the grazing cattle compared to the housed. Moreover, miR-126-5p, which is endothelial-specific and associated with aerobic exercise [39], did not show any change between cattle groups or time points in this study. These results suggest that the grazing of cattle did not cause damage of tissues such as muscles and blood vessels. Rather than tissue damage, changes in c-miRNA composition in the grazing cattle might contribute to the elevated activity of their physiological adaptation, as indicated in the extracted KEGG pathways such as endocytosis, focal adhesion of cells, axon guidance of neurons, intracellular signaling related to cell growth and/or differentiation, especially in the muscles and nerves.

### Exercise-related miRNAs: miR-148a, miR-221

In the present study, miR-148a showed a temporarily higher circulation level at 1 mo in the housed cattle than in the grazing cattle. In previous studies, miR-148a expression was up-regulated during mouse adipogenesis [41] and shown to promote myogenesis of C2C12 myoblasts [42]. The circulating level of miR-148a decreased from baseline levels after 12 weeks of endurance training [43]. Thus, the lower level of circulating miR-148a in the grazing cattle might affect expression of target genes in their skeletal muscle or adipose tissue.

The pattern of changes in the miR-221 expression level was closely similar to that of miR-148a in the present study. In previous studies in humans, the circulating miR-221 level was elevated by a single bout of exhaustive cycling or rowing training exercise [29], and was down-regulated immediately after an acute exercise bout [43]. These observations suggest that circulating miR-221 level could depend on various factors including exercise intensity and duration. MiR-221 contributes to vascular remodeling, an adaptive process involving phenotypic and behavioral changes in vascular cells in response to vascular injury with its effects on vascular smooth muscle cells and endothelial cells as well as miR-222 [34]. The lower miR-221 expression in the grazing cattle at the initial grazing stage might imply enhancement of angiogenesis in the grazing cattle following movement during grazing, compared to the housed cattle.

### Grazing-induced miRNAs: miR-19b, miR-150, miR-223, miR-320a, miR-361

No exercise-induced changes in circulating miR-19b, miR-150, miR-320a, or miR-361 have ever been reported, which may indicate the specificity of changes in those miRNAs to the grazing movements of cattle. MiR-19b and miR-361 are expressed in bovine adipose tissue. Both are annotated as relevant components in the network of core miRNAs in adipose tissue [44], suggesting the release of both miRNAs from adipose tissue. The miR-19b expression showed temporary elevation in the housed cattle at 1 and 2 mo. In addition, potential target genes of miR-19b were significantly annotated to molecular biological pathways of endocytosis, focal adhesion, axon guidance, and Wnt signaling. Taken together, circulating miR-19b might suppress genes associated with intake of microvesicles, cell adhesion and structural changes, neural network formation, and intracellular signaling at the cells miR-19b reached, more effectively in the housed cattle than in grazing cattle. Expression of miR-361 was shown to be altered by treatment with palmitate and oleate in mouse muscle C2C12 cells [24]. Therefore, miR-361 might be associated with changes in fatty acid content at the potential miR-361 recipient cells less in grazing cattle than in the housed cattle.

The miR-150 is unique in that its circulation level was down-regulated in grazing cattle at the later stage (at 4 and 7 mo) of the grazing experiment compared to that of the housed cattle. It also regulates genes related to maturation of T-cells [45] and promotes the differentiation of stem cells into megakaryocytes rather than erythrocytes [46, 47]. Transcriptomic analysis of miR-150 distribution shows that miR-150 is enriched in the abdominal and rump subcutaneous fat [48] and thymus [49] of cattle, and the spleen in mice [50]. Although we did not analyze miR-150 expression in any of those tissues, miR-150 expression in those tissues might be changed by grazing. A reduced level of circulating miR-150 in the grazing cattle could contribute to enhancement of T-cell development at the miR-150 recipient cells, resulting in the enhancement of the immune system in the grazing cattle.

There was temporary up-regulation of miR-223 in the housed cattle. This miRNA is enriched in the liver and is used as a biomarker for patients with hepatocellular carcinoma and chronic hepatitis [51]. In addition, miR-223 is also considered as an inflammatory miRNA due to its specific expression in the haematopoietic system [52]. A high expression level of miR-223 is seen in cells of myeloid lineage, with granulocytes showing the highest levels. Although the roles of miR-223 in human exercise or the grazing of animals remains unknown, our observation is similar to the result of down-regulated miR-223 level in circulation at 1 h post-exercise [43]. The temporary lowering of circulating miR-223 in the grazing cattle might indicate haematopoiesis in those cattle.

Expression of miR-320a is ubiquitously distributed in tissues of cattle [53]; however, its role remains unclear. Down-regulation of the miR-320a level is associated with up-regulation of the muscle-type phosphofructokinase gene and an increase in lactate, namely, enhancement of glycolysis. In the present study, the miR-320a level in circulation was higher in the housed cattle than in the grazing cattle at 4 mo. The lower level of glycolysis-related miR-320a in circulation [54] might be associated with muscle phenotypic changes induced by grazing as shown previously [16].

### MiR-451 changes in skeletal muscle and circulation

Expression of miR-451 is highly expressed in skeletal muscle [55] and is up-regulated in low responders to endurance resistance in humans [36]; however, the effect of human exercise or animal grazing on the circulating miR-451 level has not yet been studied. In the present study, miR-451 expression in the BP muscle of the grazing cattle was temporarily up-regulated at 2 mo compared to the housed cattle, which suggests the positive effect of grazing on miR-451 expression in skeletal muscles, as well as on miR-206 and miR-208b [16]. Intriguingly, this change synchronized with the higher level of circulating miR-451 in the grazing than in the housed cattle. According to the results, the increased level of circulating miR-451 might be caused by the release of the increased intramuscular miR-451. In addition, the higher circulation level of miR-451 might reflect a response for erythroid maturation [56] to supply more oxygen against increased movement, as shown in the restricted expression of miR-451 to erythropoietic cells in mice and human.

### Changes in circulating muscle-specific miRNAs during grazing

We hypothesized that movement during grazing affects secretion of miRNA-loading exosomes similarly to myokines, as shown in studies focusing on the effects of acute or chronic exercise in humans [29–32, 39]. Muscle-specific miRNAs are likely to be released into circulation by exercise or movement, since skeletal muscles are the major organs in the animal body and can be stimulated to release extracellular factors such as myokines by their contracting action. Indeed, muscle-specific miR-1, miR-133a/b, miR-206, and/or miR-208b in circulation have been shown to be changed by muscle-damaging downhill walking [31] and marathon running in humans [39, 57]. On the other hand, these muscle-specific miRNAs and miR-499 are present at a very low level in the serum of healthy humans who have not just exercised [30, 39].

In the present study, among the 231 exosomal miRNAs detected in the cattle plasma, muscle-enriched miR-486 and a trace of miR133b were detected, but miR-1, miR-133a, miR-206, miR-208b, and miR-499 were not detected. The difference between human exercise studies and the present cattle grazing studies can be simply explained by the difference in exercise type, duration, and intensity. Although miR-206 and miR-208b expression in the BP muscle of cattle was up-regulated by grazing [16], the unchanged muscle-specific miRNA levels in circulation in our study suggest that grazing is a mild form of exercise or movement for cattle that does not induce muscle damage.

It is noticeable that the circulation level of miR-486, a muscle-enriched miRNA, was much higher in the housed cattle at 1 mo than in the grazing cattle. Circulating miR-486 level decreases in response to both acute and chronic exercise in young men, and the changing ratio of miR-486 showed a negative correlation with VO_2 max_ [30]. The difference in miR-486 expression between human studies and this bovine study would be due to difference between species or exercise mode. The lower level of the miR-486 compared to housed cattle during the early stage of grazing may indicate a decrease in miR-486 secretion from muscle cells or an increase in the uptake from circulation, although no difference in miR-486 level in the BP muscle between the cattle was observed at 2 mo [16]. The role of circulating miR-486 in grazing cattle needs to be further investigated.

### Potential effect of environmental stress on circulating miRNAs

It is possible that the grazing conditions such as heat and humidity put environmental stress on the grazing cattle in the present study. A study investigating the effect of heat stress on bovine c-miRNAs found reduced levels of miR-150 and miR-223 and elevated levels of miR-19b and miR-320a in circulation in heat-stressed Holstein cows [58]. Indeed, the major GO terms enriched by the target genes of the changed c-miRNAs were stress- or immunity-related [58]. The immune response in heat-stressed cattle was also observed by the elevation of the neutrophil-to-lymphocyte (N/L) ratio, a known stress marker, in the blood of cattle [59]. Meanwhile, in the present study, circulating levels of miR-19b, miR-150, miR-223, and miR-320a were temporarily lower in the grazing cattle than in the housed, suggesting that there might be some stress on the grazing cattle. Nevertheless, the significant GO terms from the target genes of changed c-miRNAs in our grazing study were related to metabolism, protein phosphorylation, gene regulation, signaling, angiogenesis, blood vessel development, and neuron differentiation but not immunity or stress (S2 Table). Taken together, our results suggest that the grazing of cattle in the present study was not associated with stress related to the immune system as it was in the heat-stressed cattle.

## Conclusion

In the present study, we found that totally 231 miRNAs were present in plasma exosomes of Japanese Shorthorn (JSH) cattle. Of these c-miRNAs, circulation levels of miR-19b, miR-148a, miR-150, miR-221, miR-223, miR-320a, miR-361, and miR-486 were significantly down-regulated in the grazing cattle compared to housed cattle, whereas the miR-451 level was higher in the grazing than in the housed cattle. Synchronous miR-451 expression was also observed in skeletal muscle, which might result in secretion or intake of the miRNA between circulation and tissue cells in the grazing cattle. Functional annotation results suggested that the down-regulated c-miRNAs might suppress their target genes and affect molecular biological events associated with endocytosis, focal adhesion, axon guidance, and a variety of intracellular signaling pathways for cell growth and differentiation in grazing cattle, especially in the muscles.

## Materials and Methods

### Animals

The animals were cared for as outlined in the Guide for the Care and Use of Experimental Animals established by the Animal Care Committee of the School of Veterinary Medicine at Kitasato University, and this committee approved the study. Six JSH steers, aged 21 to 28 months and weighing 474.3 ± 45.0 kg, were raised solely on grass from the pastures of the Yakumo Experimental Farm, Kitasato University [16]. The steers were randomly divided into two groups: 3 cattle in the housed group and 3 in the grazing group.

### Sample preparation

Blood samples were drawn from the jugular vein of each animal and the plasma was prepared as 0.1% EDTA, followed by storage at -80°C until use. The blood samples were collected at 0, 1, 2, 4, and 7 months (mo) in a 7-month experimental period (Fig 1). BP muscle samples were taken from the middle part of the muscle [60] by surgical biopsy at 0, 2, 4, and 7 mo in the experimental period (Fig 1). In the first 4 months from early June to late October, the grazing cattle were fed on the pasture, whereas the housed cattle were fed in a free-stall barn with grass harvested every morning, so that the average total digestible nutrients (TDN) and crude protein (CP) contents did not differ between the groups. In the last 3 months, from late October to the following mid-January, both groups were fed with grass silage in the free-stall barn. The cattle were not fed any grain throughout the feeding, resulting in 100% fodder self-sufficiency. The muscle biopsy procedure was as follows: the animal was locally anesthetized by an intramuscular injection of 0.06 mg/kg of xylazine (Bayer, Tokyo, Japan) and a subcutaneous injection of 400 mg of lidocaine (AstraZeneca, Osaka, Japan); subsequently, a 3- to 5-cm incision was made in the skin overlying the BP muscle. All samples were rapidly transferred in RNAlater solution (Ambion, Austin, TX) and stored at -20°C until analysis.

### Exosome preparation

Ten ml of the plasma sample was mixed with 20 ml of PBS and centrifuged at 1,200 *g*, 4°C for 20 min. The supernatant was centrifuged at 12,000 *g*, 4°C for 45 min, and further centrifuged at 110,000 *g*, 4°C for 120 min. The precipitation was suspended in PBS and centrifuged at 110,000 *g*, 4°C for 70 min. The final precipitation was resuspended in PBS, stored at 4°C for a few days, and then processed for RNA preparation.

### Negative staining images by transmission electron microscope

The exosome samples were absorbed to formvar-film-coated copper grids (400 mesh) and were negatively stained with 2% phosphor tungstic acid solution (pH 7.0) for 30 sec. The stained samples were observed by a transmission electron microscope (JEM-1400 Plus; JEOL Ltd., Tokyo, Japan) at an acceleration voltage of 80 kV. Digital images (2,048 × 2,048 pixels) were taken with a CCD camera (VELETA; Olympus Soft Imaging Solutions, GmbH, Münster, Germany).

### Detection of exosome marker by immunoblot

Plasma exosome samples were mixed with Laemmli sample buffer, then boiled for 3 min and loaded onto a 12.5% polyacrylamide gel (BioRad, Hercules, CA, USA). The gel was run with Laemmli running buffer under denaturing conditions at a constant voltage of 200 V and then transferred to a PVDF membrane at 2 mA/cm^2^ for 1.5 h (25 mM Tris-HCI pH 8.3, 192 mM glycine and 15% (V/V) methanol). After transfer, the membrane was reacted with anti-CD9 antibody (MEM-61), followed by processing with ECL prime Western Blotting Detection System (GE Healthcare UK Ltd, Buckinghamshire, England) according to the manufacturer’s protocol. The chemiluminescence of CD9 bands on the membrane was detected using a FluoroChem Digital Imaging System (Alpha Innotech Corp., San Leonardo, CA, USA).

### RNA preparation

Total RNA including miRNA was extracted from muscle or plasma exosome samples using the mirVana microRNA isolation kit (Ambion) for microarray analysis of plasma samples according to the manufacturer’s protocols. The muscle sample for reference in this RNA quality test was prepared from the *semitendinosus* muscle of a Japanese Black steer according to the method described above. The quantity and quality of the RNA were determined by Agilent Bioanalyzer 2100 with an RNA 6000 Pico Kit (Agilent Technologies, Santa Clara, CA, USA). Muscle total RNAs for PCR analysis of ribosomal protein L7 (RPL7), an internal control, was prepared using ISOGEN (Nippon Gene, Tokyo, Japan).

### Microarray analysis

Three exosomal RNA samples for each feeding treatment (grazing or housed) and time point (0, 1, 2, or 4 mo) were mixed together and applied to custom microarray SurePrint G3 8x60K (Agilent) corresponding to miRBase release 19. The signals of hybridized probes were detected with Agilent Feature Extraction 10.7.3.1 using an Agilent Microarray Scanner (Agilent) and globally normalized to 90 percentile using GeneSpring GX (Agilent). Unsupervised hierarchical clustering of the miRNAs was conducted with cluster 3.0 (http://bonsai.hgc.jp/~mdehoon/software/cluster/manual/index.html) using the log-transformed data of 131 miRNAs whose (1) normalized values in the grazing cattle before grazing (at time 0) were between 2- and 0.5-fold of those of the housed cattle, and (2) raw signal values at time 0 were > 30.0. The result was visualized with Java Treeview (https://www.princeton.edu/~abarysh/treeview/) by further normalization of 1-, 2-, and 4-mo values with that of the time 0 in grazing or housed cattle so that the signal intensity of time 0 equaled 1.0 for all miRNAs in both groups of cattle. Array data were deposited in the National Center for Biotechnology Information (NCBI) Gene Expression Omnibus (GEO) database, and are accessible through GEO Series accession number GSE69717 (http://www.ncbi.nlm.nih.gov/geo).

### cDNA synthesis

The synthesis of cDNA was done from 250 ng of total RNA for muscle samples or 9 μl of the final product of RNA preparation for exosome samples, with the miScript II RT kit (Qiagen, Tokyo, Japan) at 37°C for 60 min, and then the enzyme was inactivated at 95°C for 5 min. The cDNAs for muscle samples were synthesized from 1,000 ng of total RNA by ReverTra Ace qPCR RT kit (Toyobo).

### Quantitative PCR (qPCR) analysis

Quantitative PCR was performed using the CFX96 thermal cycler (Bio-Rad, Hercules, CA) under the following program: 15 min at 95°C, followed by 40 cycles of 15 s at 95°C and 30 s at 60°C. For plasma samples, qPCR was performed using the Thunderbird SYBR qPCR kit (Toyobo, Tokyo, Japan) in combination with the miScript Primer Assay for let-7g, miR-19b, miR-29b, miR-30a, miR-30d, miR-103, miR-126-5p, miR-144, miR-148a, miR-150, miR-155, miR-221, miR-223, miR-320a, miR-361, miR-425-5p, miR-451, miR-486, miR-489, miR-1249, and miR-2888 (Qiagen) according to the manufacturer’s protocol. For muscle samples, qPCR was performed using the Quantitect SYBR Green PCR Kit (Qiagen) in combination with RPL7 PCR primers [16]. Differences in the expression ratios of the target miRNA/let-7g for plasma samples and of the target miRNA/RPL7 for muscle samples were compared between the grazing periods as well as between the cattle groups [16]. Melting curve analysis was used to confirm the specificity of the amplification reactions.

### Prediction and Functional Annotation of miRNA Target Genes

The miRNA target genes were predicted using the TargetScan system (Release 6.2, http://www.targetscan.org/) [61]. To classify the target genes according to functional annotation, both GO and pathway analysis were performed on the target genes of c-miRNA differentially expressed in grazing and housed cattle based on qRT-PCR results. In this study, DAVID bioinformatic resources (version 6.7, http://david.abcc.ncifcrf.gov) [62, 63] were applied to the potential target genes with setting *bos taurus* as the background species, to enrich characteristic KEGG pathway terms defined by KEGG (http://www.genome.jp/kegg/) for the respective miRNA-mediated biological process. Extraction of the terms was considered significant when the Bonfferoni-adjusted *P*-value was < 0.05.

### Statistical analysis

The expression data are shown as means ± SDs and were compared by statistical analyses at a significance level of *P* < 0.05. The statistical analyses among time points of the experimental periods were carried out by one-way repeated-measures ANOVA using js-STAR 2012 software (ver. 2.0.6j; http://www.kisnet.or.jp/nappa/software/star/index.htm), followed by multiple comparisons between every two time points by multiple comparison with Holm’s method. The comparisons between the cattle groups were carried out by two-sided Student’s *t*-test for each time point.

## Supporting Information

S1 Fig Changes in exosomal let-7g level in plasma of grazing and housed cattle analyzed by microarray. Solid and broken lines are of grazing and housed cattle, respectively. The values of grazing and housed cattle at 1, 2, and 4 mo were normalized by those at 0 mo, respectively.
(PPTX)

S1 Table Raw and normalized expression data of microRNAs in bovine plasma exosomes in microarray analysis. Samples were collected at 0, 1, 2, and 4 mo from grazing cattle (G-1, G-2, G-3, G-4) and (H-1, H-2, H-3, H-4).
(XLSX)

S2 Table Gene ontology terms significantly (Bonfferoni *P*-value < 0.05) extracted from potential target genes of down-regulated circulating microRNAs in grazing cattle. (XLSX)
