# Supplementary figures and images for: Grazing Affects Exosomal Circulating MicroRNAs in Cattle
Source: PLoS One. 2015 Aug 26;10(8):e0136475. doi: 10.1371/journal.pone.0136475 (PMC4550388; doi:10.1371/journal.pone.0136475)

## Slide 1
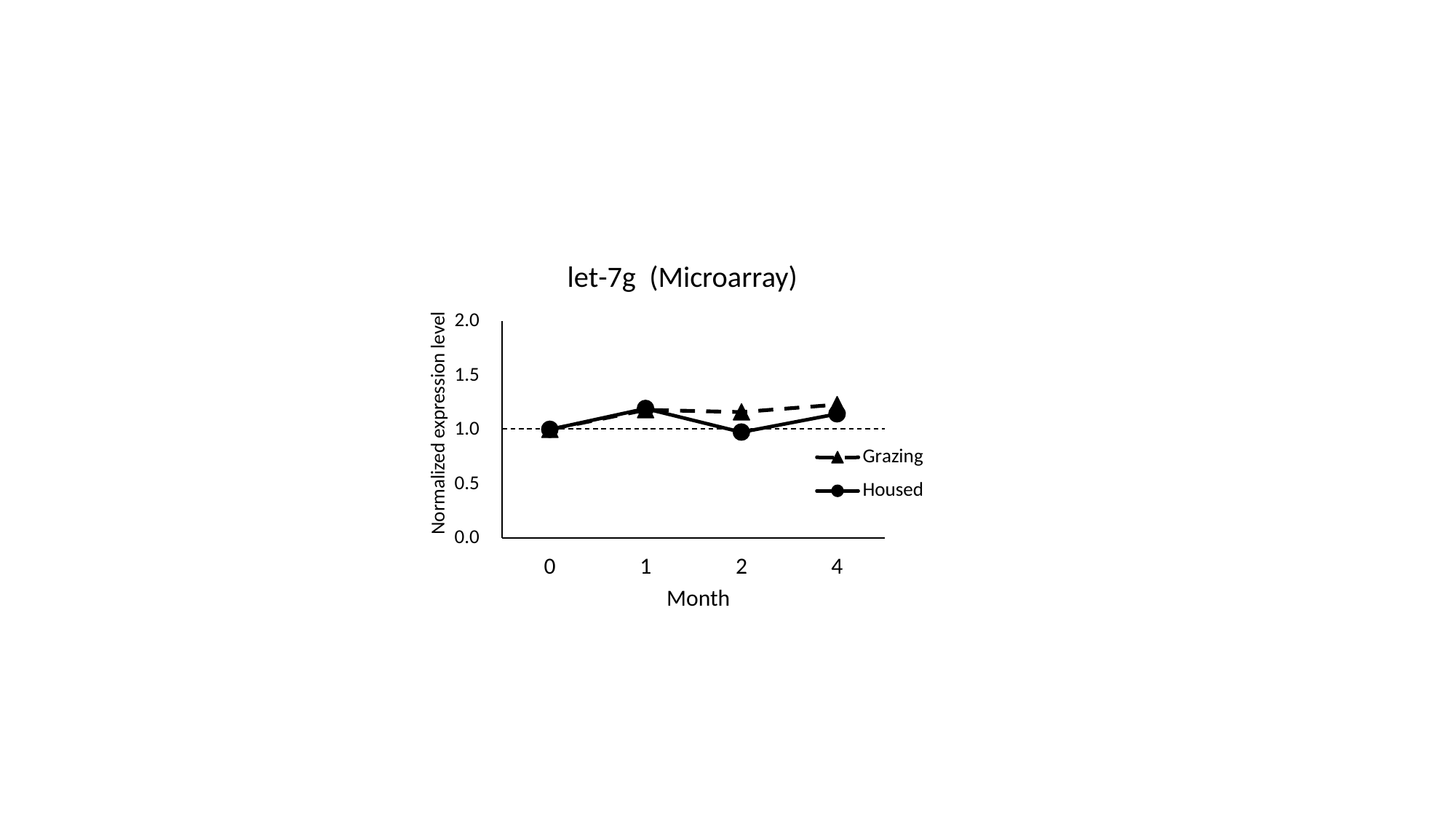

Supplement: S1 Fig — Solid and broken lines are of grazing and housed cattle, respectively. The values of grazing and housed cattle at 1, 2, and 4 mo were normalized by those at 0 mo, respectively. (PPTX) [file pone.0136475.s001.pptx]
